# Globoids and Phytase: The Mineral Storage and Release System in Seeds

**DOI:** 10.3390/ijms21207519

**Published:** 2020-10-12

**Authors:** Claus Krogh Madsen, Henrik Brinch-Pedersen

**Affiliations:** Section of Crop Genetics and Biotechnology, Department of Agroecology, Aarhus University, Forsøgsvej 1, 4200 Slagelse, Denmark; ClausKrogh.Madsen@agro.au.dk

**Keywords:** phytate, phytase, globoids, nutrient storage, protein storage vacuole

## Abstract

Phytate and phytases in seeds are the subjects of numerous studies, dating back as far as the early 20th century. Most of these studies concern the anti-nutritional properties of phytate, and the prospect of alleviating the effects of phytate with phytase. As reasonable as this may be, it has led to a fragmentation of knowledge, which hampers the appreciation of the physiological system at hand. In this review, we integrate the existing knowledge on the chemistry and biosynthesis of phytate, the globoid cellular structure, and recent advances on plant phytases. We highlight that these components make up a system that serves to store and—in due time—release the seed’s reserves of the mineral nutrients phosphorous, potassium, magnesium, and others, as well as inositol and protein. The central component of the system, the phytate anion, is inherently rich in phosphorous and inositol. The chemical properties of phytate enable it to sequester additional cationic nutrients. Compartmentalization and membrane transport processes regulate the buildup of phytate and its associated nutrients, resulting in globoid storage structures. We suggest, based on the current evidence, that the degradation of the globoid and the mobilization of the nutrients also depend on membrane transport processes, as well as the enzymatic action of phytase.

## 1. Introduction

Seeds contain a sporophyte embryo and nutrient storage tissues enclosed within a protective coat. The stored nutrients serve to facilitate the establishment of the plant until it can acquire sufficient nutrition from its surroundings. Carbon in the form of starch and lipids, as well as nitrogen in the form of storage proteins make up the bulk of the seed’s reserves, but a full complement of nutrients is needed. Phosphorous is mainly stored as phytate (myo-inositol 1, 2, 3, 4, 5, 6 hexakisphosphate), and is remobilized by a specific class of phosphatases: phytases. Phytate and phytases have attracted research interest mainly because of the antinutritional properties of phytate, and the ability of phytases to alleviate the negative effects of phytate (reviewed in [1]). Here, we focus on the system from a plant physiological perspective, and review the way in which phytate is the central molecule in a storage structure—the globoid—that stores a number of elements besides phosphorous, and is formed in a highly regulated manner and broken down by phytase during germination.

## 2. Phytate

**Figure 1:**
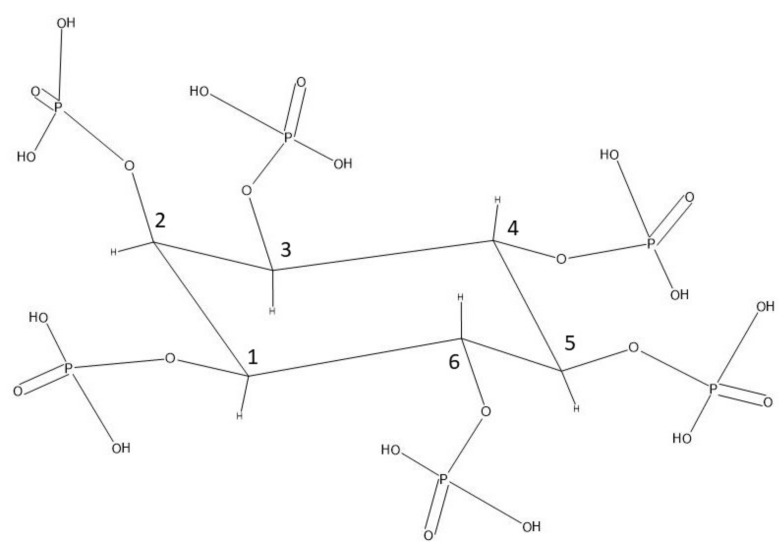
Phytate molecule in the 1ax/5eq conformation. Redrawn in ACD/ChemSketch after [3].

Phytate is hexaphosphorylated inositol, and its most important chemical properties derive from the six phosphate groups. Each phosphate group has two titratable hydrogen ions, 12 in all, with pKa values ranging from 1.9 to 9.5. Between 8 and 9 of the 12 sites are in a deprotonated state at physiological pH [2]. Phytate adopts the 1ax/5eq conformation at pH 0.5 to 9.0 (Figure 1) [3]. The phosphate groups are strong chelators of various cations. The chelates may be soluble at low pH and in phytate excess, whereas insoluble phytate salts are frequently formed above approximately pH 6 and in an excess of di- or trivalent metal ions. At pH 7.4, sodium phytate forms complexes with metal cations in the following decreasing order: Cu^2+^, Zn^2+^, Ni^2+^, Co^2+^, Mn^2+^, Fe^3+^, Ca^2+^ [4]. This is largely in agreement with Torres et al. (2005), who found the order Al^3+^, Fe^3+^, Cu^2+^, Zn^2+^, Mg^2+^, Fe^2+^, (Ni^2+^, Mn^2+^), Cd^2+^, Ca^2+^, Co^2+^ for the octo-deprotonated phytate ion (the positon of Fe^3+^ and Co^2+^ differs between the two investigations) [5]. Alkaline earth phytates precipitate as penta-cation salts, whereas alkaline phytates are fully soluble [6]. The titrations suggest that divalent metals in general precipitate as penta-cation phytates [4]. In addition, proteins may precipitate with phytate. As opposed to metal phytates, proteinaceous precipitates are promoted by a low pH, and also depend on the intrinsic properties of the proteins, such as the isoelectric point [7].

### Biosynthesis

Phytate biosynthesis starts with the conversion of glucose 6-phosphate to inositol 3-phosphate (Ins(3)P1) by the enzyme myo-inositol (3) P1 synthase (MIPS). This reaction is the sole source of the inositol backbone. From Ins(3)P1, the synthesis can proceed to phytate by two routes: the phosphatidyl inositol pathway and the lipid independent pathway.

The phosphatidyl inositol pathway starts with the dephosphorylation of Ins(3)P1 to yield myo-inositol, which is then joined with a lipid moiety by phosphatidylinositol synthase to form phosphatidyl inositol (PtdIns). PtdIns is then phosphorylated at first the fourth and then then the fifth inositol carbon by specific kinases in order to produce PtdIns(4,5)P2. The inositol phosphate moiety is released by phospholipase C to produce Ins(1,4,5)P3. The product is further phosphorylated by a 6-/3 kinase to produce Ins(1,3,4,5,6)P5. The final step to phytate is accomplished by a 2-kinase [8].

The lipid independent pathway may start from myo-inositol through the action of myo-inositol kinase (MIK), or directly from Ins(3)P1. Maize (*Zea mays* L.) MIK activity provides Ins(1/3)P1 and Ins(4/6)P1, as well as a trace amount of Ins(5)P1 [9]. The pathway proceeds to Ins(1,3,4)P3 by the action of unknown kinases. From here, inositol tris/tetrakisphosphate kinase (ITPK) advances the synthesis to Ins(1,3,4,5,6,)P5, and the last step is completed by a 2-kinase, as for the PtdIns pathway [10].

Phytate biosynthesis is believed to occur in the cytoplasm with subsequent transport to the protein storage vacuole (PSV) [11,12]. This view is supported by the discovery of the responsible transporter, a multidrug resistance-associated protein (MRP) ATP-binding cassette (ABC) transporter, which is responsible for the low phytic acid 1 (lpa-1) allele in maize [13]. The silencing of orthologs has been shown to cause low phytic acid phenotypes in soybean (*Glycine max* L.), *Arabidopsis thaliana*, common bean (*Phaseolus vulgaris* L.) and rice (*Oryza sativa* L.) [13,14,15,16]. The *Arabidopsis* ortholog *AtMRP5* was shown to be a specific and ATP dependent phytate transporter in a yeast recombinant assay. Furthermore, AtMRP5-GFP located to the tonoplast [14]. Work on the MRP–ABC phytate transporter demonstrated not only the cytosolic origin of phytate but also that cytosolic phytate concentrations are tightly regulated, since phytate does not accumulate in the cytosol instead of the PSV. This seems to be accomplished by a combination of negative feedback to the biosynthetic genes and the degradation of the synthesized phytate [15]. Cytosolic conditions permit only up to 49 µM phytate in solution as the penta magnesium complex. Above this concentration, the magnesium salt would precipitate [17]. Calcium and other divalent ions would also precipitate, along with magnesium, presumably rendering all but micromolar cytoplasmic phytate concentrations toxic.

## 3. Globoids

**Figure 2:**
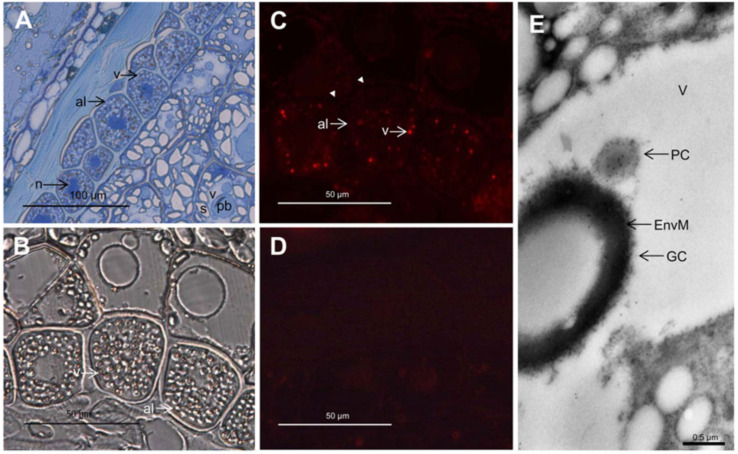
Light (**A**–**D**) and immunoelectron (**E**) microscopy analysis of the localization of PAPhy in the developing wheat grain, approximately 18 days post anthesis. (**A**) Toluidine blue-stained semithin cross-section of endosperm, aleurone, and pericarp tissues. (**B**) Differential interference contrast microscopy with indications of the PSVs. (**C**) Immunofluorescence detection of PAPhy in 1 µm thick sections. The aleurone vacuoles are clearly labeled, while there is no fluorescence from any other compartment of the cell, the apoplast (arrowheads), or other cell types. (**D**) Immunofluorescence of a 1 µm thick section incubated with secondary antibody only. There is virtually no background from the secondary antibody. (**E**) Immunoelectron microscopy analysis showing an aleurone PSV with gold labeling of the protein crystalloid. al, Aleurone; EnvM, globoid enveloping membrane; GC, globoid crystal; n, nucleus; pb, protein body; PC, protein crystalloid; s, starch; v, vacuole. Reprinted in part from [25]. Copyright: American Society of Plant Biologists; http://www.plantphysiol.org.

Globoids are spherical inclusions characterized by their high content of phytate. They are often lost when preparing samples for microscopy, either being dissolved during fixation or torn from the sample during sectioning because of their hard and brittle character. The resulting void is called the ‘globoid cavity’. Thicker sections or carefully optimized sample preparation are necessary to observe the globoids directly. Barley globoids stained red with toluidine blue in mild acidic solution which enabled the distinction between globoids and other subcellular structures [18]. However, this could not be reproduced by later investigators [19]. Glutaraldehyde/osmium fixed globoids appear amorphous and electron-opaque when subjected to electron microscopy [18]. An example electron micrograph is shown in Figure 2E. The most reliable ways to identify globoids in microscopy are those that allow the simultaneous detection of their elemental composition, in which case the globoids are revealed by their phosphate content. Examples of such procedures are discussed in the following sections.

Globoids form in PSVs in tissues that accumulate phytate in high levels. They are surrounded by a membrane with some of the hallmarks of a lytic vacuole. Thus, they can be described as a vacuoles within vacuoles [20]. The presence of the globoid membrane has been controversial, as many investigators have failed to identify it. This can be explained by fixation procedures that were not optimized for the experimental tissue. Performing the fixation at a lower pH was sufficient to detect the globoid membrane in soybean [21].

With the exception of a few species discussed below, and some indirect evidence, it is unknown which proportion of the total phytate is sequestered into globoids. The complete silencing of the *lpa1* encoding the MRP–ABC transporter in maize reduces grain phytate by 93% [13]. This suggests that >90% of phytate in storage tissues is transported to vacuoles. This is also the location of the globoids, but it should be noted that phytate may also exist in the vacuolar lumen.

The PSV of some species may also contain another inclusion: the protein crystalloid or protein/carbohydrate body. This inclusion is made clearly distinct from the globoid by its electron density, histochemical staining and the lack of membrane [22].

### 3.1. Cereal Globoids 

The distribution of phytate in small grain cereals differs from what is seen in maize. In rice, the pericarp and aleurone was found to contain 80% of the total seed phytate. For wheat (*Triticum aestivum* L.), 87% was found in the aleurone. The remaining phytate in rice and wheat is almost exclusively found in the germ (embryo). Maize, on the other hand, contains 88% of its phytate in the germ. The remainder is mainly found in the endosperm (it is not clear if the endosperm included the aleurone) [23]. More recently, 95.2% of maize phytate was determined to be present in the embryo [24].

The phytate-rich tissues also contains globoids. Barley (*Hordeum vulgare* L.) globoids are found in the protein storage vacuole of the aleurone cells, and there are one to three globoids per vacuole of up to three micrometers in diameter [18]. A similar arrangement exists in wheat [19,25]. In addition to the aleurone globoids, wheat has smaller globoids in the scutellum, and to some extent in other parts of the embryo [26]. Globoids purified from wheat bran ranged in size from 1.5–5 µm [27]. Rice contains globoids in the aleurone layer, and in the scutellum and embryo tissues. The embryo globoids are smaller than those of the aleurone and scutellum [28,29]. Maize has globoids in the scutellum and aleurone layers. The scutellum globoids varied in size, but were often 1.5–2 µm in diameter [24].

Purified globoids from rice contained 48% phytate, 12% protein, 8% non-phytate carbohydrates, and 15% moisture. The major metallic elements were K (9%), Mg (8%) and Ca (0.4%). Zn, Fe, Cu and Mn were present at <0.1% [30]. Purified globoids from wheat contained 40 % phytate, 46% protein, 10% moisture and the metallic elements K (7.6%), Mg (3.2%) and Ca (0.43%). Zn, Fe, Cu, Mn and Na were present at <0.1 %. In addition to phosphorous and metallic elements, wheat globoids contained sulphur (0.17%) and trace amounts of boron (1.3 ppm) [27]. Both studies used the same purification procedure, which involve non-aqueous density gradient centrifugation to purify the globoids. The sum of the components found in purified wheat globoids somewhat exceeds 100%. One possible explanation for this is the semi-quantitative nature of the Bradford assay that was used to quantify the protein component [31].

Further insight into the role of the globoids as mineral storage structures is provided by imaging technologies that examine the globoids in situ with more or less invasive sample preparations. Some technologies can investigate the mineral distribution across a whole seed section, whereas others permit the investigation of individual globoids. The distribution and speciation of metals in wheat grains were investigated by synchrotron X-ray fluorescence and extended X-ray absorption fine structure (EXAFS). The iron concentrations were highest in the aleurone layer near the embryo, and on the dorsal side of the seed, in the crease region as well as in the embryo, specifically in the scutellum, coleoptilar ring and coleorhiza. Manganese, Cu, Ni and Zn were largely distributed in the same manner as iron in the aleurone, except for the elevated levels found in the crease region for Mn, Cu and Zn. Zinc and Cu were more prevalent in the embryo compared with the other metals. EXAFS was used to investigate the speciation of iron and zinc in the aleurone. Iron was octahedrally coordinated by six oxygen atoms and one or two phosphorous atoms, suggesting that iron is bound to one or two phosphate groups. Zinc was tetrahedrally coordinated by the oxygen atoms of one or two phosphate groups. Thus, both metals were likely in complex with phytate in the aleurone layer [32]. A similar investigation was undertaken using diamond blade-cut sections. Again, the highest concentrations of Fe was found in the aleurone, scutellum and nucellar projection (crease region). The aleurone is notably more iron rich on the dorsal side and the intracellular distribution resemble that of the globoids. The speciation of iron was further investigated by X-ray absorbtion near-edge structure (XANES) imaging. This showed that Fe was phytate/citrate bound in the aleurone, and phytate bound in the scutellum and modified alurone of the crease region. Furthermore, Cu and Zn reached the highest concentrations in the aleurone and co-localized with P, suggesting that they are phytate bound [33]. Similar results demonstrating the co-localization of Mg, Fe, Zn, Na and Al with P in the aleurone cells of re-hydrated, cryosectioned wheat grains were obtained by synchrotron soft X-ray microscopy [34]. Furthermore, K, Mg, Ca, Mn, Fe, Zn were detected in wheat globoids from the aleurone and scutellum by energy dispersive X-ray microanalysis (EDX) [35]. Globoids from the scutellum were richer in Ca and Mn, but contained less Fe and Zn compared to globoids from the aleurone.

Rice grains were examined by EDX. As expected from the analysis of purified globoids, globoids in situ were enriched in Mg, K and Ca compared to background levels. The study revealed some differences in globoid composition between the tissues. While calcium was present in aleurone globoids, it was absent from globoids in most parts of the embryo. Iron and zinc, on the other hand, were absent from aleurone globoids, but were present in the mesocotyl, coleoptile and radicle. In addition, zinc was found in scutellum and plumule globoids [29]. The time course of the accumulation and localization of P, K, Ca, Fe, Zn and Cu in rice grains was investigated by synchrotron-based x-ray microfluorescence (µXRF). The elements follow a similar trend of accumulation for the first 15–20 days after flowering (daf), except for Ca, which peaks around 10 daf but shows little variation throughout grain filling. Phosphorous, K, Ca and Fe were mainly found in the aleurone throughout grain filling and in mature seeds. There was some overlap between Zn and P, but much of the Zn was located deeper in the seed, perhaps in the subaleurone layer. Copper had an even more pronounced localization to the subaleurone layer.

Furthermore, maize globoids from the scutellum were examined by EDX. Phosphorous, K and Mg were the major elements detected. Calcium, Zn and Fe were present in trace amounts. Aleurone globoids contained comparatively more Ca [24].

### 3.2. Legume Globoids

The major crop legumes—soybean, peanut (*Arachis hypogaea* L.), faba bean (*Vicia faba* L.), common bean, and peas (*Pisum sativum* L.)—form globoids in the PSV of the cotyledons. These globoids are small and rare compared to, e.g., those found in the aleurone layer of small grain cereals. There are also examples of legumes with large and frequent globoids; *Cassia artemisioides* and *Clianthus formosus* [36], marama beans (*Tylosema spp*.) [37,38] and *Bauhinia spp*. [39]. It has been suggested that the ratio of Mg and Ca to K is responsible for a modest tendency to globoid formation in the major legumes. Thus, it is assumed that a higher proportion of K would render the phytate more soluble and result in an even distribution in the PSV. Indeed, the authors did successfully increase the globoid formation in pea cotyledons by injecting the pod with a solution of 0.1 M MgCl_2_ and 0.1 M Ca(NO_3_)_2_. However, we suggest that the smaller and less frequent globoids may result in part from the distribution pattern. Cereals and legumes are not very different with respect to their phytate content and the ratio of total P to phytate P (Table 1). However, the major legumes store phytate in the cotyledons which occupy the largest volume of the seed, whereas cereals exclude phytate from the largest seed tissue: the starchy endosperm. Phytate is therefore distributed in a much larger proportion of the legume seeds, so the reduced size and frequency of the globoids should be expected. It is also a distinct possibility that the distribution of phytate between globoids and the PSV lumen differs among species. As such, Prattley and Stanley (1982) found that only 10%–15% of soy phytate was deposited in the globoids. The remainder was found in the vacuole lumen [40]. Peanut globoids, on the other hand, contained 50% of the phytate, and the remainder was in the PSV lumen [41].

The phytate ABC transporter, first identified in maize, is also critical for phytate accumulation in soybean [13] and common bean [15]. This suggests that key elements of the phytate storage system are conserved between cereals and legumes. As for cereals, K and Mg are the major cations of legume globoids, with the occasional detection of Ca [36]. Purified globoids from soybean contained 23.8% phytate, 3.6% K, 1.6% Mg and 0.9% Ca [40]. This is approximately half the phytate content of the cereal globoids discussed above, and proportionally the same amount of the associated metals (Table 2). It should be noted, however, that the soy globoids were isolated by filtration using an aqueous buffer (0.5 M Tris pH 10), whereas the cereal globoids were isolated using a non-aqueous procedure. Peanut globoids were divided into fractions of large 2–4 µm and small <2 µm globoids, which differed in composition. Large globoids contained 7.7% phytate, 50.7% protein, 12% moisture and 0.5% oxalic acid. The metal content was K 2.5%, Mg 1.8%, and trace amounts of manganese. Small globoids contained 28% phytate, 35.1% protein, 8.6% moisture and 3.5% oxalic acid. The metal content of the small globoids was K 2.0%, Mg 2.5%, Ca 0.5%, and trace amounts of manganese. As for soybean, the peanut globoids were isolated by an aqueous procedure [41].

The *lpa-1* mutation affecting the phytate transporter in common bean is associated with a ‘hard to cook’ (HTC) phenotype, which is caused by the redistribution of calcium in response to reduced phytate [50]. This suggests that phytate has an important role as a storage compound for calcium in legumes, even though it is not always detected in the individual globoids examined, e.g., by EDX. It is possible that phytate in the PSV lumen, rather than the globoids, counteracts the HTC phenotype. 

### 3.3. Arabidopsis Globoids

The model plant *Arabidopsis thaliana* contains globoids in the aleurone, protoderm, ground meristem and procambium of the cotyledons, as well as the shoot- and radicle apex. They contain P, K, Mg and Ca, and traces of Zn and Fe, but virtually no Mn. Comparatively abundant Ca appears to be a novelty of *Arabidopsis* globoids [51]. The highest content of iron was found in the globoids of the procambium, but the main site of iron accumulation was later shown to be the vacuoles of the endodermis [52]. This iron is sequestered into globoids, and requires at least one of the transporters AtNRAMP3 and AtNRAMP4 for its successful remobilization during germination. AtNRAMP4 was localized to the tonoplast and the globoids themselves (presumably to the globoid membrane) [48]. The iron content in globoids from the endodermis has not been quantified to our knowledge. The Arabidopsis PSV structure was studied in dry, ‘stratified’ (2 days in an aqueous buffer at 4 °C in the dark) and ‘germinated’ (stratified seeds incubated in light at 21 °C for 24 hours) seeds [53]. Structures identified as globoids inside the PSV were decorated by NRAMP4 and γ-TIP antibodies in the dry seeds, with some remnants being present in the stratified seeds. The γ-TIP antibodies also labelled a larger compartment inside the PSV of the germinating seeds, whereas NRAMP4 antibodies decorated punctuate structures in the cytosol at this point. The tonoplast of the PSV, on the other hand, was decorated by α-TIP antibodies throughout the experiment. Since α-TIP and γ-TIP are considered markers of PSV and lytic vacuoles, respectively, this supports the view of globoids being contained in a lytic-like vacuole within the PSV. This was further corroborated by the detection of the vacuolar H + ATPase (V-ATPase) and vacuolar H+-pyrophosphatase (V-PPase) in the same membranes as γ-TIP. Furthermore, the compartment inside the PSV of germinating seeds was found to be acidic by Neutral Red staining. The formation of the internal lytic compartment was further investigated by antibodies against markers associated with the pre vacuolar compartment (PVC), Syp21 and mRab. They too labled the internal lytic compartment, punctuate sites in the cytosol, and pearl chain-like punctuate sites in the periphery of the lytic compartment and connecting the lytic compartment with the exterior of the PSV. This is strong evidence for trafficking from the golgi apparatus to the lytic compartment. The authors conclude that the globoids and the internal lytic compartment that appear during germination are distinct, because NRAMP4 and γ-TIP labeling almost disappear from internal structures in the PSV during stratification, and only γ-TIP re-emerges in the internal lytic compartment [53]. Thus, the study leaves some intriguing questions for future research about the fate of the globoid, including its membrane, in the early phase of germination, and about the origin of the internal lytic compartment.

### 3.4. Globoids in Other Species and Non-Seed Tissues

Tobacco (*Nicotiana tabacum* L.) and tomato (*Solanum lycopersicum* L.) seeds were used to investigate the globoid membrane [20].

Globoids were isolated from canola (*Brassica napus* L.) seeds. Here, the globoid fraction was rich in cruciferin and napin-like storage proteins. Two non-storage proteins were identified: myrosinase-binding protein and the ‘Brassicaceae PSV-embedded protein’ BPEP. Homologs were subsequently identified in Arabidopsis [54]. Both proteins have uncertain functions, and are properly restricted to the Brassicaceae, yet they offer a rare insight into the protein component of globoids.

Large (2.3–5.6 µm) globoids were detected in flax seeds (*Linum usitatissimum* L.), and could be recovered in ash following slow thermal oxidative degradation. The minerals K, Mg, Mn, Ca and Fe co-localized with P in the globoids [55].

Globoids have also been found in poplar (*Populus alba* L.) twigs during winter, where they appear to function as a seasonal P reservoir. As is the case in seeds, the twig globoids form inside PSVs [56]. 

Globoids have also been reported in the pollen of *Lilium longiflorum* and *Chlorophytum elatum*, both of which are members of the Liliaceae. The lily family pollen globoids are, to our knowledge, the only globoids which are not found inside vacuoles. Instead, they are found in compartments described as storage bodies or vesicles by the authors, respectively [57,58].

### 3.5. Globoids in Low Phytate Crops

Several authors have investigated the fate of globoids, and the distribution of minerals normally associated with globoids, in low phytate crops. The maize mutant *lpa1-1* has a 62% reduction in phytate. This translates directly into a reduction of globoid size from 1.5–2 µm to less than 0.8 µm in the scutellum. The globoids gained at tendency to aggregate, and aleurone globoids would sometimes be non-spherical. There was little difference or redistribution in the mineral content of the *lpa1-1* seeds compared to the wild type. The most notable differences were 1/3 higher iron content and an 18% reduction in calcium [24]. Similar results were obtained with wheat showing a 38% phytate reduction [35]. The mineral content and distribution was investigated in four barley lines with phytate reductions between 26 and 94%. Again, there was remarkably little difference in the mineral content and distribution, except for a higher iron content in the most phytate-reduced line [59].

### 3.6. In Summery

Phytate is the major storage compound for phosphorus in seeds, and its accumulation is associated with the occurrence of globoid structures. Globoids show considerable plasticity in terms of their size, composition and distribution in the seed (Table 1 and Table 2). Nevertheless, there are some features which appear to be universal. Globoids are always found inside a PSV, or in highly similar structures in the novel case of lily family pollen globoids. Seed globoids are, in all probability, always surrounded by a membrane.

They always contain phytate, but protein is generally more abundant. Very little is known about the protein component. They also invariably contain metal cations. Magnesium and potassium are always present, whereas the content of other metals varies between species and tissues. The evidence shows that globoids are major storage structures for P, K, Mg and Ca in seeds, supported by a pool of phytate in the PSV lumen. The chelated ions are rendered temporarily inert as part of the solid globoid structure. This allow cells with globoids to accumulate ions in high amounts without compromising the cells’ ionic homeostasis. The molar ratios of the major cations to phytate in globoids, as deduced from the papers discussed in this section, are given in Table 2. The large globoids of peanut appear to be oversaturated with cations, but it should be noted that they contain oxalate as an additional anion. Rice globoids are also slightly oversaturated, but not more so than might be explained by the accuracy of the data. The remaining globoid samples were associated with metallic cations corresponding to six to eight positive charges. The alkaline earth ions always numbered at least two per phytate molecule, and potassium was roughly three, except for the peanut globoids. Therefore, we propose a model in which the core constituent of globoids is tri-potassium di-magnesium phytate (K_3_Mg_2_InsP_6_). This salt would provide a solid matrix capable of meeting cellular storage demand by immobilizing additional cations with the remaining seven titratable hydrogens of the phytate ion. It is possible that the relationship of at least two alkaline earth ions (usually magnesium) for each phytate molecule is the key to the building of a supramolecular storage structure. It has been shown that manganese and zinc can bridge phytate molecules by complexing with two at the same time [49]. If the—likewise divalent—alkaline earth ions behave in the same way, two cations per phytate molecule would allow for an extensive supramolecular structure. Further evidence for this hypothesis is provided by the use of phytate to create corrosion-resistant surfaces on metallic magnesium and its alloys. These deposits form a multideck structure where the inner layer is in direct contact with the metal, whereas the additional layers consist of magnesium phytates, which presumably adhere to the layers below and above by complexing shared magnesium ions [60].

Additional metals in the globoids are generally found in lower concentrations, which vary depending on the tissue type and position in relation to the overall seed geometry. It is noteworthy that the in vivo association of phytate and metals does not correspond to the chemical affinity of the ions or solubility of the phytate salts. This shows unequivocally that the formation of globoids is not a simple precipitation of phytate salts. It is a regulated process in which, presumably, both the PSV membrane and the globoid’s own membrane act as checkpoints. As the main site of phytate accumulation, the globoids and the PSV lumen are also, by extension, the main storage site of the inositol backbone. This should not be overlooked, because inositol and its derivatives have many important cellular functions [61]. Finally, it is clear that the globoids contain a significant amount of protein.

## 4. Phytases

Phytases are phosphatases that can initiate the de-phosphorylation of phytate. They are a prerequisite for the remobilization of phytate bound P, and must be assumed to be ubiquitous in the plant kingdom as phytate. Phytases are also widespread in the microbial world, and even animals may express phytase. The first report which coined the term ‘phytase’ was Suzuki et al. (1907), who reported the purification of the enzyme from rice bran, and made observations of the formation of ‘phosphoric acid’ (inorganic phosphate) during the germination of rice, barley and brassicas [62]. Since then, numerous plant phytases have been biochemically purified, and the hydrolysis of phytate to release inorganic phosphorus during germination has become well established. In the following, we will focus on plant phytases of known amino acid or nucleotide sequence.

### 4.1. Classification

Phytases can be classified based on their stereospecificity and phylogenetic kinship. The International Union of Pure and Applied Chemistry and the International Union of Biochemistry and Molecular Biology recognize three classes of phytase based on the first phosphate position they hydrolyse: EC-3.1.3.8 (3-phytases), EC-3.1.3.26 (4-phytases) and EC-3.1.3.72 (5-phytases). This follows the recommended numbering of carbon atoms in the myo-inositol backbone (D nomenclature). However, the alternative L nomenclature is often used in the literature, in which case they are 1-, 6- and 5-phytases, respectively [47]. It should be noted that a D-6 phytase from *E. coli* has been reported. 3-, 4- and 5 phytases have been reported in plants [63].

The phytase classes histidine acid phytases (HAP), β-Propeller phytase (BPP), Purple acid phosphatase phytase (PAPhy) and cysteine phosphatase-like phytase (CP-phytase) have been described based on phylogenetic kinship. The classes BPP and CP-phytase are only known from microorganisms, whereas HAP and PAPhy phytases have been described in plants. The structure and properties of microbial HAPs have been extensively studied due to the economic importance of these enzymes. Briefly, HAPs fold into two domains: the α, and the larger α/β domain. The active site is made up of the conserved RHGXRXP and HD motifs, where the histidine of the first motive and the aspartate of the HD motif take direct part in the catalytic reaction [64]. Purple acid phosphatases are metalloproteins which contain a dinuclear Fe^3+^ Me^2+^ center in the active site. The divalent ion can be Fe^2+^ (mammalian PAPs), Zn^2+^, or Mn^2+^ (plant PAPs). The ions are chelated by seven amino acid residues (D, D, Y, N, H, H, H). These seven metal-ligands are contained in a shared pattern of five common consensus motifs (DxG/GDx2Y/GNH(E,D)/Vx2H/GHxH) [65,66]. Plant PAP phytases (PAPhy) contain an additional four consensus motifs. The structural basis for their possible involvement in phytase activity remains to be clarified [25].

### 4.2. Plant HAPs

A phytase was biochemically purified from maize, and was used to raise rabbit antibodies [67]. Screening a maize phage display library with this antibody yielded the first plant phytase nucleotide sequence (*PHYT I*): a HAP [68]. The subsequent screening of a maize genomic library using the cDNA clone as a probe provided two highly homologous genes (*PHYT I* and *PHYT II*) [69]. The two genes were shown to have an overlapping expression pattern, with *PHYT I* having the highest expression level. After 1 day of imbibition, *PHYT I* and *II* mRNA accumulated in the coleoptile, coleorhiza and radicle of the seeds. No signal was detected in the scutellum or embryo leaves. A lower, but consistent, signal remained detectable in the roots after one month. The immunolocalization of the proteins revealed a similar pattern [69]. PHYT-I was expressed in *E. coli*. Both native and recombinant proteins migrated as dimers with an apparent molecular weight of 76 kDa during native polyacrylamide gel electrophoresis, but the recombinant protein did not show any phosphatase activity [68].

Given the amount of plant genome sequence data amassed over the past two decades, it may be expected that orthologs had been identified in many other plants. However, we and others have been unable to find such reports in the literature, and blasting the PHYT-I amino acid sequence against the uniprot database returns no obvious candidates. Apart from PHYT-II, the best matches were a gene of approximately 50% amino acid identity reported in several *Oryza spp*., and a wheat gene of 46% amino acid identity. These genes remain uncharacterized. Thus, it seems that *PHYT I* and *II* are maize novelties, or that they are at least highly diverged from their othologs in other plants. 

Subsequently identified plant HAPs belong to the subfamily of Multiple Inositiol Polyphosphate Phosphtases (MINPP or MIPP). This group was first described in animals as phytases and inositol phosphate phosphatases involved in cellular signaling. They are phylogenetically distinct from the microbial HAPs, and generally possess a KDEL-like ER-retention signal. MINPP-like sequences were also identified in plants [70]. More recently, MINPP were also identified in bacteria [71]. The first plant MINPP to be identified as such and characterized was the lilly pollen alkaline phosphatase [72]. Earlier biochemical work revealed some unusual properties for this phytase: an alkaline optimum (pH 8), dependence on calcium and inhibition by EDTA, 5-phytase specificity, and a high substrate specificity for phytate [73,74,75].

Four wheat and three barley MINPP cDNA clones were isolated. The encoded proteins were more than 90% identical. Being MINPP’s, the barley and wheat proteins showed some notable differences compared to the lily pollen enzyme. Recombinant wheat and barley MINPP have an acidic optimum of pH 4.5, with a sharp decrease above pH 5. Furthermore, they are expressed in the grains during development, as well as germination [76].

### 4.3. Plant PAPhys

The first plant PAPhy was biochemically purified from the cotyledons of ten day old soybean seedlings. The enzyme had a pH optimum between 4.5 and 5.0 and high affinity for phytate when compared to ATP, polyphosphate and *para*-nitrophenyl phosphate (pNPP). The N-terminal and four internal peptides were sequenced by Edman degradation, thus facilitating a PCR-based cloning strategy [77]. An ortholog, *MtPHY1*, was isolated from *Medicago truncatula* and used in a transgenic strategy to improve the phosphate acquisition of *A. thaliana*. The native function of *MtPHY1* was not investigated [78]. More recently, a root associated *PAPhy* from another legume, *Stylosanthes guianensis*, was characterized, and was shown to be involved in phosphate acquisition [46]. A phytase was purified from tobacco root extrudates, and was identified as a PAPhy by mass spectroscopy. The enzyme had a pH optimum of 5.0 to 5.5, and a broad substrate activity with the catalytic efficiency towards deoxyribonucleotide triphosphates, ATP and pNPP being higher than towards phytate [45].

The *A. thaliana* genome encodes 29 PAPs [79]. Two of these have been reported to possess phytase activity. The first, *AtPAP23*, is expressed in flowers, and shows a moderate activity towards phytate of 35% compared to pNPP [80]. The other, *AtPAP15*, is ubiquitously expressed. The recombinant proteins expressed in yeast with a histidine tag, and in *E. coli* with a GST tag, both showed high activity towards phytate, exceeding the activity towards pNPP. The *E. coli-*expressed protein with the GST tag removed had a pH optimum between 4.5 and 5.0. The overexpression and, conversely, knock out, of *AtPAP15* increase and decrease foliar ascorbate, respectively. The authors suggested that AtPAP15 exerts its effect on ascorbate by providing a substrate for the inositol-ascorbate biosynthetic pathway [81]. Another report found a much lower efficiency towards phytate compared to pNPP for AtPAP15 purified from transgenic tobacco plants. However, insertion line knock out plants had a reduction in seedling phytase activity of 35 to 55% of the wild type. Promoter-GUS plants provided further insights in the expression pattern of AtPAP15. Its expression in all of the organs was confirmed, but it was not evenly distributed. A high expression was seen in the emerging radicle of two day old seedlings. A strong expression was also observed in the roots, hypocotyl and cotyledons in the following days. Mature plants stained in the vasculature of the roots and leaves, and in pollen grains [82]. The expression during germination and in pollen grains is consistent with a role in the mobilization of phytate-bound nutrients, whereas the expression associated with the vasculature suggests that the enzyme may have multiple roles. The leaf and root expression of a PAPhy, *PtPAP3* from trifoliate orange, was upregulated upon P starvation [83].

Two phytases were biochemically purified from wheat bran. Both were found to have the N-terminal sequence EPAXTLTGPSRPV [84]. In retrospect, this was the first evidence for PAPhys in wheat. Another group disclosed nucleotide sequences in a patent application shortly after [85]. To our knowledge, the inventors did not pursue a peer reviewed publication. Further biochemical evidence for the presence of PAPhy in wheat was provided using the PAP-specific inhibitor molybdate and western blotting with an antibody raised against AtPAP15 [86].

A comprehensive study of cereal PAPhys was undertaken by Dionisio et al. (2011). A total of nine cDNAs were cloned from wheat (4), barley (3), maize (1) and rice (1), as well as additional non-phytase PAPs. The wheat and barley transcripts could be grouped as ‘a’ and ‘b’ isoforms, based on the timing of their expression and C-terminal sequence. The ‘a’ isoforms were predominantly expressed during grain filling, and matched the N-terminal sequence reported previously for wheat bran phytase. The ‘b’ isoforms were predominantly expressed during germination, and the maize and rice *PAPhys* behaved in essentially the same manner as the ‘b’ isoforms [25]. The isoforms were later shown to originate from a single set of paralogs residing on Triticeae chromosome 5 (*PAPhy_a*) and 3 (*PAPhy_b*). The causative gene duplication happened between the divergence of the Triticeae from rice and *Brachypodium*, respectively; thus, the presence of both paralogs is restricted to a small subset of grasses, of which the only cereals belong to the Triticeae, e.g., wheat, barley and rye. The presence of multiple transcripts of each paralog in wheat is caused by the allohexaploid nature of the genome [87]. The three *PAPhy_a* homeologs in wheat remain more similar to the *PAPhy_a* genes of their respective ancestors than to each other [88]. Barley *PAPhy_a* is almost exclusively responsible for the phytase activity in the mature grain (MGPA) [89]. High MGPA is only found in cereals with the *PAPhy_a* gene; thus, we can infer that *PAPhy_a* is also responsible for the high MGPA in wheat and rye. This is also supported by the expression and biochemical data discussed above. For a discussion of this, and the implications for human and animal nutrition, we refer to our recent review [90]. The biochemical properties of the cereal PAPhys were investigated using the recombinant proteins expressed in *Pichia pastoris*. Cereal PAPhys have a strong preference for phytate over 10 potential substrates, including pNPP and ATP (based on Kcat/Km). The pH optimum was 5.5 for TaPAPhy_a1 and 5.0 for TaPAPhy_b1 (wheat ‘a’ and ‘b’ types respectively) [25]. The rice *OsPAPhy_b* (termed *OsPHY1* by the authors) was also characterized by Li et al. (2012). The expression peaked in the seeds after four days of germination, but was high during all of the days (2–10). A pH optimum of 3.5 was determined for the enzyme expressed in *E. coli* [91]. Wheat PAPhy_a was localized in developing grains by light and immunoelectron microscopy. Light microscopy showed punctuate labeling in the PSVs of the alurone layer. The labeling at the ultrastructure level was mostly focused on the vacuolar particles interpreted as protein crystalloids, rather than globoids (Figure 2) [25]. This places the PAPhy_a enzyme remarkably close to its substrate at a time when the substrate is still accumulating rather than hydrolyzed. There are no known reversible proteinaceous inhibitors of phytases. Instead, plants appear to rely on proteases, e.g., to counter pathogen-secreted phytases [92]. This suggests that the activity of PAPhy_a is tightly controlled by the chemical environment, likely the pH, and/or localization.

### 4.4. In Summary

Plant phytases can be PAPhys, MINPPs or, rarely, HAPs, which are closer to fungal HAPs than the MINPPs. Plant phytases are commonly expressed during seed germination, but expression may also occur during grain filling. The *PAPhy_a* gene of the Triticeae cereals is expressed during grain filling, and remains latent in the seeds, resulting in the high MGPA of this group of cereals. The accumulated enzyme is stored in close proximity to its substrate, and is set to hydrolyse it when the conditions during germination permit. It is not known with certainty if *PAPhy_a* also takes part in the metabolism of phytate during grain filling. A comparison of the phytate accumulation in the Triticeae with that of rice (which lack *PAPhy_a*) suggests this is not the case. The accumulation is similar. Nevertheless, phytate can be hydrolysed during grain filling, as demonstrated by the work on MRP–ABC phytate transporter mutants discussed in Section 2.1. This process cannot be attributed to *PAPhy_a,* because it occurs in species lacking this gene. Furthermore, the process is clearly cytosolic. Thus, it is more likely to be carried out by MINPPs, which are also known to be expressed during grain filling.

Phytases are also frequently associated with roots, and are sometimes extrudated from the roots. Some phytases have broader expression patterns that suggest that they may be acting on other substrates. There is no clear functional division between the phytase families in plants.

## 5. Phytate, Phytase and the Globoid Form a Nutrient Storage and Release System

In the previous sections, we reviewed phytate chemistry and biosynthesis, the globoid and its cellular context, and plant phytases. Together, these components form a nutrient storage and release system that is, to the best of our knowledge, ubiquitous in angiosperms.

**Figure 3:**
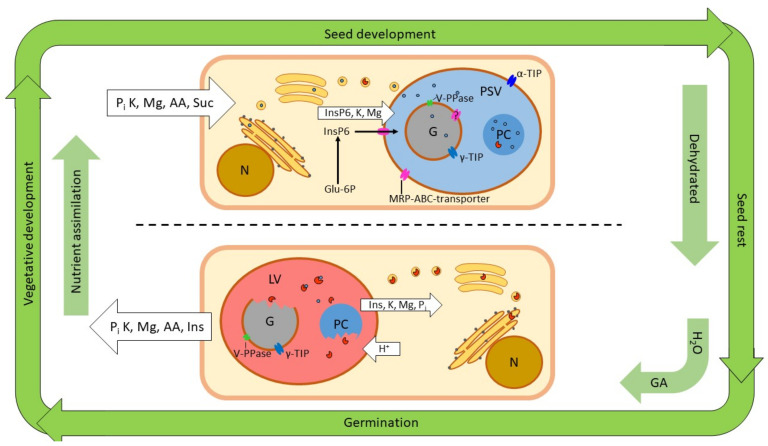
Events in a storage tissue cell in relation to the plant’s life cycle. Above the dotted line is a cell during seed development. Below it is a cell during germination. The organelles shown are the nucleus (N), with the attached rough ER and the golgi apparatus above. The protein storage vacuole (PSV) with neutral pH is indicated by the blue color, and the lytic vacuole (LV) with acidic pH is indicated by the red color. Only one vacuole is shown in each cell for graphic simplicity. The vacuoles contain the globoid (G) and the protein crystalloid (PC). Ion and molecule fluxes are indicated on the white arrows; phytate biosynthesis and transport is indicated during seed development. The blue dots are storage proteins, and the red circles missing a sector are hydrolases.

Plants assimilate nutrients from the soil and through photosynthesis during vegetative growth. As the seeds develop, they receive nutrients which accumulate in the storage tissues that typically make up the bulk of the seeds. When phosphorus arrives in the storage tissue cells it is converted to phytate by one of two biosynthetic routes operating in the cytoplasm. It is then transported to the protein storage vacuole by a specific MRP–ABC transporter (Figure 3). A proportion of the phytate enters the globoid envelope, where it participate in the formation of the solid globoid structure. It is not known how phytate enters the globoid envelope, but the membrane is known to accommodate tonoplast proteins, so the MRP–ABC transporter likely transports phytate across this membrane as well. Metal ion nutrients, principally potassium and magnesium, also arrive to the storage tissue cell. Potassium is actively transported across the tonoplast by specific antiporters [93]. Again, we can speculate that the same transporters might facilitate transport to the final destination of potassium: the globoid. Furthermore, magnesium is known to be transported across tonoplast membranes by antiporters [94]. It is an intriguing possibility that Mg^2+^ ‘hitchhikes’ on phytate to be transported through the MRP–ABC-transporter as a phytate chelate. To our knowledge, it remains to be investigated whether the transporter can accommodate such a complex.

Furthermore, nitrogen, in the form of amino acids, arrives at the storage tissue cell. The amino acids are used to synthesize storage proteins, which traffic to the PSV via the secretory pathway. Some hydrolases may also be synthesized and stored in the PSV (e.g., the PAPhy, a phytase of the Triticeae cereals). The seeds are dehydrated towards the end of their development, and the starchy endosperm (but not the alurone layer) of monocots undergoes programmed cell death (PCD) [95].

Germination commences when the seeds are rehydrated, the temperature is right, and species-specific requirements to break dormancy have been fulfilled. Gibberellin is produced in the embryo and serves as the hormonal signal, which coordinates the germination events, including the mobilization of the stored nutrients. One of the responses to the GA signal is the acidification of the PSV. For instance, the pH in the PSV of barley aleurone protoplasts decreased from 6.6 to 5.6 in response to external GA. The reaction was detectable after two hours, and was essentially completed after six [96]. This acidification is key to the PSV transition into a lytic organelle, as it allows for the activity of a multitude of acidic hydrolases (Figure 3). These include proteases that hydrolyze storage proteins, and phytases that act on phytate [22,97]. The first few days of germination also see extensive morphological changes to vacuolar membrane structures. The multiple PSVs, typically found in storage tissue cells, merge to form one central vacuole [98,99]. Direct observations of globoids during germination are rare. Bolte et al. (2011) observed globoids in ‘stratified’ *Arabidopsis* seeds imbibed in the dark and at 4 °C. However, the globoids were gone after 24 hours at 21 °C [53]. We suggest that the rehydration of the cell, and the acidification of the PSV and the globoids themselves, cause the globoids to rapidly dissolve. The acidification of the globoids themselves is supported by the presence of vacuolar V-PPase in the globoid membrane [20]. The authors even suggest that phytate itself may drive the proton pump, because phytate can be pyrophosphorylated. The detection of pyrophosphorylated phytate (PP-InsP_6_) in barley aleurone cells by Brearley and Hanke (1996) lends support to this hypothesis [100]. More recently, the occurrence of PP-InsP_6_ was studied in *Arabidopsis,* and the presence in *Camelina sativa* L., cotton (*Gossypium hirsutum* L.) and maize was also confirmed [101]. The synthesis of PP-InsP_6_ can occur through an auxiliary catalytic activity of ITPK, which also participates in phytate biosynthesis (see Section 2.1). PP-InsP_6_ is receiving increasing attention as a signal molecule in plants; for instance, in phosphate sensing [102]. The cofactor requirements of V-PPase further support a close association with the globoid content. Mg is necessary for the activity of mung bean V-PPase, and K stimulates the activity [103]. It is therefore possible that the remobilization process is self-propelled, and accelerates with the dissolving globoid, providing more and more substrate for the V-PPase. The globoid membrane also harbors γ-TIP, an aquaporin, which facilitates the influx of water to dissolve the contents [20]. One possible explanation for the rapid disappearance of the globoids is therefore that accelerating acidification dissolves the contents and causes an osmosis-driven influx of water through γ-TIP, eventually bursting the membrane or causing it to fuse with the tonoplast.

An additional observation, suggesting that globoid degradation requires more than phytase enzymes, is the absence of globoids in the starchy endosperm of cereals. This tissue contains starch grains and protein bodies that are hydrolyzed by amylase and proteases secreted from the aleurone, so why not also globoids? The answer may be that globoid degradation requires something that the dead cells of the endosperm cannot offer, such as active transmembrane pumps.

Once dissolved (or simultaneously), phytate is hydrolyzed by phytases. The hydrolysis is a slow process compared to the rapid disappearance of the globoids. The process was studied in wheat, barley, rye and oat (*Avena sativa* L.), with more than half of the initial phytate still being present after 96 h [104]. Similar results were obtained with barley and rye, although with a faster hydrolysis in rye, with less than half of the phytate remaining after three days [105]. Furthermore, rice and soybean had significant amounts of phytate left after several days of germination [106,107]. The aleurone layer of wheat and barley commenced PCD four days after imbibition, suggesting that living cells are not needed to complete the phytate hydrolysis after this point [95].

## 6. Conclusions

The nutrient storage- and release system that has phytate and phytases as central components is ubiquitous in angiosperm seeds, and variants of it may be found in other species and tissues. It assembles during seed development in a regulated manner, relying on compartmentalization and specific membrane pumps and transporters. The composition of the globoid storage structure is only superficially understood, and warrants further investigation; for instance, with respect to the protein composition and the possible occurrence of lipids, carbohydrates and small molecules which may have been overlooked in earlier investigations. The transport processes during globoid synthesis are another major theme for future research. It is not known whether the globoid membrane contains its own unique transporters for the import of cations and phytate, or if it uses the same system as the tonoplast. The mobilization of the nutrients is no less complex, and is also not fully understood. It seems to rely on a combination of enzymatic activities (principally phytase) and proton pumps. Future focus on these components may help our overall understanding of the release of phytate-associated nutrients. A starting point would be to document the steps in the disappearance of the globoids in early germination.

## Figures and Tables

**Table 1:** Phytate content and the proportion of the total phosphorous of selected seeds according to Eeckhout and De Paepe (1994) [42], Viveros et al. (2000) (values in ()) [43], Steiner et al. (2007) (values in []) [44] and Nagy et al. (2009) (marked with *) [14]. The main storage tissue and size of the globoids is given as discussed in Section 3.1, Section 3.2, Section 3.3, Section 3.4 and Section 3.5.

	Phytate, % of Seed Mass	Phytate, % of Total P	Main Tissue	Globoid Size
Rice	0.23	72	Aleurone	
Wheat	0.22 (0.29) [0.23]	67 (73) [45]	Aleurone	1.5–5 µm
Maize	0.19 [0.18]	68 [46]	Embryo	1.5–2 µm
Barley	0.22 (0.26) [0.19]	60 (63) [47]	Aleurone	
Soybean	[0.33]	[48]	Cotyledons	
Peanut ^1^	0.32	47	Cotyledons	up to 4 µm
Peas	0.17 (0.24) [0.24]	45 (58) [44]	Cotyledons	
Arabidopsis	1.6 *	94 *		
Flax	[0.34]	[49]	Cotyledons	2.3–5.6 µm

^1^: The phytate content is for extracted peanut cake.

**Table 2:** Molar ratio of the major cations relative to phytate, and the sum of their charge relative to phytate. This was calculated from available data for purified globoids [27,30,40,41].

	K	Mg	Ca	Mg + Ca	Total Positive Charge
Rice	3.2	4.5	0.1	4.7	12.5
Wheat	3.2	2.2	0.2	2.3	7.9
Soybean	2.6	1.8	0.6	2.4	7.4
Peanut(large)	5.5	6.3	0.0	6.3	18.2
Peanut(small)	1.2	2.4	0.3	2.7	6.6
